# Natural Product-Based Hybrids as Potential Candidates for the Treatment of Cancer: Focus on Curcumin and Resveratrol

**DOI:** 10.3390/molecules26154665

**Published:** 2021-07-31

**Authors:** Nicola Micale, Maria Sofia Molonia, Andrea Citarella, Francesco Cimino, Antonina Saija, Mariateresa Cristani, Antonio Speciale

**Affiliations:** Department of Chemical, Biological, Pharmaceutical and Environmental Sciences, University of Messina, Viale Ferdinando Stagno D’Alcontres 31, I-98166 Messina, Italy; nmicale@unime.it (N.M.); mmolonia@unime.it (M.S.M.); acitarella@unimore.it (A.C.); fcimino@unime.it (F.C.); mcristani@unime.it (M.C.); specialea@unime.it (A.S.)

**Keywords:** hybrid compounds, hybrid nanosystems, curcumin, resveratrol, anticancer activity

## Abstract

One of the main current strategies for cancer treatment is represented by combination chemotherapy. More recently, this strategy shifted to the “hybrid strategy”, namely the designing of a new molecular entity containing two or more biologically active molecules and having superior features compared with the individual components. Moreover, the term “hybrid” has further extended to innovative drug delivery systems based on biocompatible nanomaterials and able to deliver one or more drugs to specific tissues or cells. At the same time, there is an increased interest in plant-derived polyphenols used as antitumoral drugs. The present review reports the most recent and intriguing research advances in the development of hybrids based on the polyphenols curcumin and resveratrol, which are known to act as multifunctional agents. We focused on two issues that are particularly interesting for the innovative chemical strategy involved in their development. On one hand, the pharmacophoric groups of these compounds have been used for the synthesis of new hybrid molecules. On the other hand, these polyphenols have been introduced into hybrid nanomaterials based on gold nanoparticles, which have many potential applications for both drug delivery and theranostics in chemotherapy.

## 1. Introduction

Despite enormous drug discovery efforts, cancer continues to be one of the leading causes of death worldwide. It currently ranks second in terms of mortality and, according to WHO reports, accounted for nearly 10 million victims in 2020 [1]. The difficulty in treating this disease is due to its multifactorial and polygenic nature, involving multiple organs, tissues, and biochemical pathways [2]. Therefore, as with many other complex diseases, the standard single drug treatment has proven to be ineffective over time. Strategies to overcome the multifactorial nature of this disease include the administration of drug cocktails (complex therapy) or drugs in a fixed dosage ratio (combined therapy) aimed at different targets to obtain a synergistic effect and reduce the spread of drug resistance [3]. Nevertheless, several issues related to this mixed chemotherapy treatment of cancer, such as the possibility of unforeseen drug–drug interactions, poor patient compliance, insurgence of severe side-effects, and non-selectivity toward cancerous cells, remain unresolved [4]. In the last few years, the combination strategy shifted swiftly to the “hybrid strategy”, namely the design of a new molecular entity (the hybrid molecule) containing two or more biologically active molecules [5]. The term “hybrid” has been invariably used as a synonym by the scientific community to identify a codrug (two identical or different pharmacophores/drugs joined by means of a cleavable linker), a fused drug (the same as a codrug but joined without a linker), a conjugate (bioactive compound(s) covalently linked to a functional molecule), a chimeric drug (two or more pharmacophores/drugs merged in a new chemical entity), and a multifunctional/multimodal/multipotential (and so forth) drug [6]. Furthermore, the hybrid strategy (hence the term hybrid) implies that this new therapeutic entity has superior features compared with the individual components (e.g., toxicity and bioavailability). Based on this last (and more important) consideration, the term “hybrid” has been further extended to nanotechnology, with special regard to the development of drug delivery systems in which the bioactive component(s) are embedded or conjugated to biocompatible nanomaterials. The hybrid nanosystems are generally classified on the basis of the structural components (e.g., inorganic/organic building blocks), accessory moieties (e.g., crosslinkers, stimuli-responsive moieties, and antibodies), and the architecture of hybridization (e.g., nanoclusters, nanoparticles, nanotubes, nanoshells, nanorods, nanocrystals, nanocages, etc.) [7]. The hybridization process can take place at the level of the delivery platform, or it can be directly carried out by coating the nanomaterial with bioactive compounds. The use of these smart nanomaterials represents a new trend of pharmaceutical research, as they may offer a plethora of advantages compared with mere drug therapy, including the possibility of achieving targeted chemotherapy with specific release to cancer cells and metastasis sites [8,9].

Naturally occurring products have been used for the treatment and prevention of a variety of chronic pathologies, including cancer, and there is an increased interest in plant-derived compounds due to the necessity to find alternative sources of new drugs with beneficial health properties. In particular, polyphenolic compounds, one of the most diverse groups of plant secondary metabolites, are known for their health-promoting properties and have attracted attention as promising antitumor agents and also because, generally, they are able to act as multifunctional agents. Thus, nanoscale formulations have been developed to ameliorate not only their pharmacodynamic profile but also to improve their solubility, stability, and bioavailability to address and overcome their limitations [10,11,12].

The aim of the present review is to report the most recent and intriguing research advances in the development of hybrids (in the broad sense of the term) encompassing the common feature of containing a plant-derived bioactive component for the potential treatment of various types of cancer. Given the vastness and complexity of this topic, as well as the large number of plant polyphenols that have been considered as potential substrates for the design of hybrid products, we focused our attention on two polyphenols, curcumin (Cur) and resveratrol (Res), which possess unique physicochemical features and are well-known for their wide spectrum of biological activities, including anticancer properties. Polyphenols may be categorized primarily into flavonoids and nonflavonoids. The basic skeleton of flavonoids (flavonols, flavones, flavan-3-ols, anthocyanidins, flavanones, and isoflavones) can have numerous substituents, and the water solubility of flavonoids increases with the presence of sugars and hydroxyl groups as substituents on the skeleton. The nonflavonoids include diverse classes of polyphenols, such as stilbenes and curcuminoids. The curcuminoid Cur and the stilbene Res have been extensively studied for their beneficial properties against human chronic diseases, such as cancer [13], and are representatives of the group of polyphenols with poor bioavailability. In fact, polyphenols can be generally classified into three categories: (1) high solubility and poor cell membrane permeability, (2) low solubility and poor cell membrane permeability, and (3) low solubility and high cell membrane permeability [14,15]. The polyphenols Cur and Res examined in the present study belong to types 2 and 3, respectively. Cur is insoluble in cold water, and Res also shows a very low solubility (about 0.03 g/L). Furthermore, Res is stable at an acidic pH, while its degradation increases exponentially at a pH above 6.8 [15]; Cur is highly susceptible to chemical degradation in alkaline aqueous solutions (pH ≥ 7.0) but can promote crystallization in aqueous systems under acidic conditions [16]. Thus, it is obvious that efforts to outcome their limits and improve their potential applications must be made.

The review is focused on two kinds of hybrid nanoproducts that appear particularly interesting because of the innovative chemical strategy involved in their development. As described in the first part of the paper, Cur and Res have been used for the synthesis of new hybrid compounds characterized by better chemico–physical and pharmacological properties compared with the parent drugs [17]. On the other hand, these two molecules can be introduced in the development of hybrid materials based on gold nanoparticles, a material endowed with extraordinary biocompatibility and physicochemical properties, and they consequently have many potential applications in the pharmaceutical industry for drug delivery in chemotherapy [18,19,20].

## 2. Chemical Features and Anticancer Effect of Curcumin and Resveratrol

**Figure 1:**
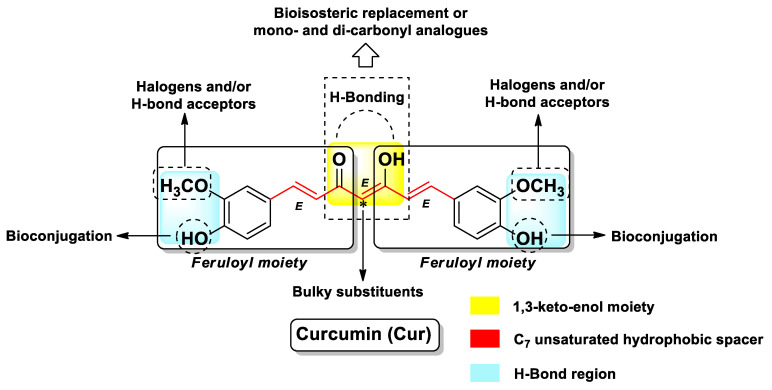
Chemical features and SAR insights of Cur.

Cur (diferuloylmethane; Figure 1) is one of the bioactive components of *Curcuma longa* Linn., and has been used for the management of multiple diseases in different cultures. Its application is limited by its low water and plasma solubility, chemical instability under alkaline conditions, poor oral absorption, and rapid metabolism. In addition, the polyphenol Res (3,4′,5-*trans*-trihydroxystilbene), a phytoalexin produced by several plant species, possesses a wide variety of biological properties that have been extensively studied both in vitro and in vivo. Unfortunately, Res, like Cur, shows low aqueous solubility and poor chemical stability, as well as low bioavailability, all factors limiting its clinical effectiveness. Both Cur and Res are claimed to possess significant anticancer properties due to their capability to act as multifunctional agents.

The imbalance between cell death and cell proliferation is one of the major factors involved in cancer development. First, the antioxidant properties of Cur and Res, because of their capability to modulate antioxidant enzymes activity, can likely contribute to the anticancer effects of these polyphenols. However, in some experimental studies on cancer cells, Cur and Res appeared able to promote massive ROS production, which could lead to cell death [21,22]. Cur and Res can inhibit cell survival signaling [23] and lead to apoptosis, not only activating the extrinsic and intrinsic pathways but also inducing cell-cycle arrest. Interestingly, there is evidence that Cur and Res can activate not just apoptosis but also autophagic cell death [24]. Furthermore, Res was shown able to induce cancer cell death by upregulating sirtuin 1 (Sirt1) [25].

The tumor microenvironment is a complex environment containing various cellular components (e.g., stromal cells, immune cells, and vascular endothelial cells), multitudinous factors (e.g., cytokines), abundant extracellular matrix (ECM), and their various cross-talk networks. Cur and Res can have modulatory effects on the tumor microenvironment [26]. In particular, Cur and Res can inhibit cancer growth through the inhibition of pathways related to epidermal growth factor (EGF) and vascular endothelial growth factor (VEGF), pro-inflammatory cytokines, and pro-inflammatory enzymes [27]. Finally, the invasion and metastasis of cancer cells involve the destruction of the ECM and basement membrane by proteolytic enzymes, such as matrix metalloproteinases (MMPs), in particular MMP-9, which have been shown to be inhibited by Cur and Res in several experimental models [25,28].

Finally, both Cur and Res were shown to play a significant role in tumor treatment through targeting microRNA (miRNA) acting as tumor suppressors and oncogenes [29], and acting as modulators of the epithelial-to-mesenchymal transition [30].

The chemical features of Cur consist of two symmetrical feruloyl moieties connected through a methylene group. Its overall structure includes a linear seven-carbon chain with a 1,6-heptadiene motif in *E*-configuration, a central 1,3-diketone component, and two external aryl moieties (Figure 1). However, computational studies have shown that the 1,3-diketone component of Cur is more stable in the 1,3-ketol-enol tautomeric form (as depicted in Figure 1) [31]. This latter feature, coupled with the unsaturated nature of the C_7_ chain with trans C=C, gives rise to a planar and linear structure for Cur with the methoxy groups pointed out in the opposite direction with respect to the 1,3-keto-enol group, according to prior crystal X-ray diffraction studies [32]. Moreover, the presence of the 1,3-ketol-enol group fosters the formation of stable metal–Cur complexes, which have been recently comprehensibly reviewed elsewhere [33]. As said before and recently reviewed by several authors [21,24,34,35], Cur has been shown to target all the main steps of cancer development, being capable of interfering with several biochemical pathways involved in the proliferation and survival of cancer cells as well as directly and indirectly binding different targets. In this view, essentially all the chemical parts of the structure of Cur are considered vital for the activity. The two feruloyl moieties play a critical role in binding to protein targets (H-bonding and π–π stacking interactions). Successful modifications include a variation of the phenyl substitution pattern, mostly halogens or other H-bond acceptors and, in a few cases, bioisosteric replacement of the phenyl group with equivalent (hetero)aryl rings. Furthermore, the two phenolic -OH groups may be exploited for the development of a wide variety of bioconjugates. The 1,3-keto-enol moiety is also very important for the biological activity of Cur, but it is also responsible for its instability in an alkaline medium (which already occurs at pH above 6.5). In order to overcome such instability problem, fruitful modification of this structural motif includes the development of mono- and di-carbonyl analogs (symmetrical and asymmetrical), heterocyclic analogs (to embed the 1,3-diketo-enol moiety), and the insertion of bulky substituents at the methylene group. The length of the C_7_ chain is normally kept unchanged, as attempts to shorten or extend it in most cases lead to less biologically active derivatives (Figure 1) [34,36].

**Figure 2:**
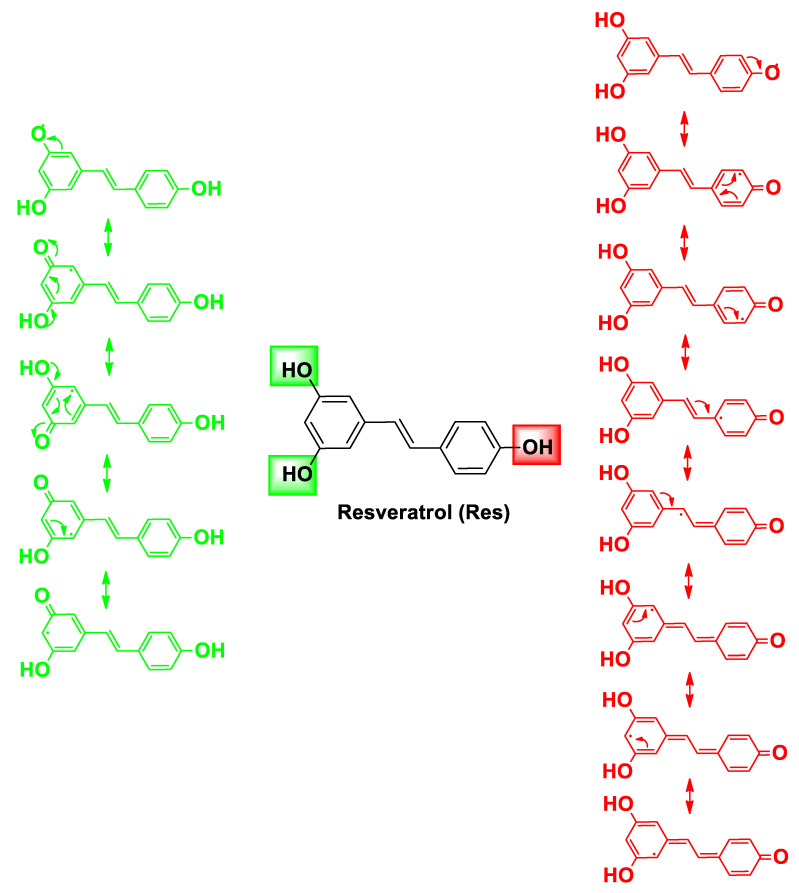
Resonance structures of the semiquinone form of Res after hydrogen abstraction of the catechol -OH (green) and phenol -OH (red).

In addition, Res, regarding its antitumor activity, is known to affect a variety of cancer stages, from initiation and promotion to progression, by affecting the diverse signal-transduction pathways that control cell growth and division, inflammation, apoptosis, metastasis, and angiogenesis [22,37,38,39,40,41]. In particular, the anticancer properties of Res seem related to its capability to modulate the cell redox status both by acting as an antioxidant and by promoting ROS production. The extraordinary antioxidant potential of Res relies on its unique structural motif that entails the presence of a double-bound C=C united to a *p*-phenol group and a resorcinol group; both aromatic groups are able to provide several resonance structures upon radicalic hydrogen abstraction of the -OH in the para and meta position, respectively (Figure 2). Therefore, modifications of this small molecule usually lead to compounds with reduced antioxidant properties, although some Res-like derivatives with improved pharmacokinetic and/or pharmacodynamic parameters have been developed. The structural changes mostly include alkylation, halogenation, glycosylation, and further hydroxylation [42,43,44].

## 3. Hybrid Compounds Containing Curcumin and Resveratrol

The synthesis of Cur- and Res-based hybrid compounds represents an innovative approach for drug discovery in the field of anticancer chemotherapy. This strategy is based on the possibility to connect, via covalent chemical bonds, two or more bioactive scaffolds endowed with pharmacological activity [6,45,46,47] so that the fusion of different pharmacophores in a new multifunctional compound is aimed to obtain a better efficacy in comparison with the parent drugs in terms of improved pharmacokinetic and pharmacodynamic properties and decreased toxicity [45] (Table 1 and Table 2).

For example, to overcome the negative physicochemical properties of Cur, some Cur-based hybrids have been projected starting from Cur monocarbonyl analogs by deleting the reactive β-diketone moiety, which is an unfavorable feature for the real bioavailability of this polyphenol (e.g., [58,59]). On the other hand, this technique can improve the interaction with specific targets critical for the regulation of a cellular process. For example, tubulin is the globular protein constituting microtubules, which play a key role in cell division. Cur-based hybrids developed by Sharma et al. [59] and Singh et al. [58] are more efficient tubulin polymerization inhibitors, targeting the Cur-binding site close to the vinblastine binding site. Yin et al. [49] developed Res–cinnamoyl derivates as new tubulin polymerization inhibitors able to target the colchicine binding site. Furthermore, the monocarbonyl ligustrazine–Cur hybrids synthesized by Ai et al. [49] are better inhibitors of thioredoxin reductase (TrxR), a fundamental enzyme for the regulation of the cell redox balance often overexpressed in cancer cells. The Res–caffeic acid-based hybrids developed by Li et al. [51] can simultaneously target both acetylated and phosphorylated STAT3, a cytoplasmic protein that plays a fundamental role in oncogenic signaling pathways, while Res targets only acetylated STAT3.

**Figure 3:**
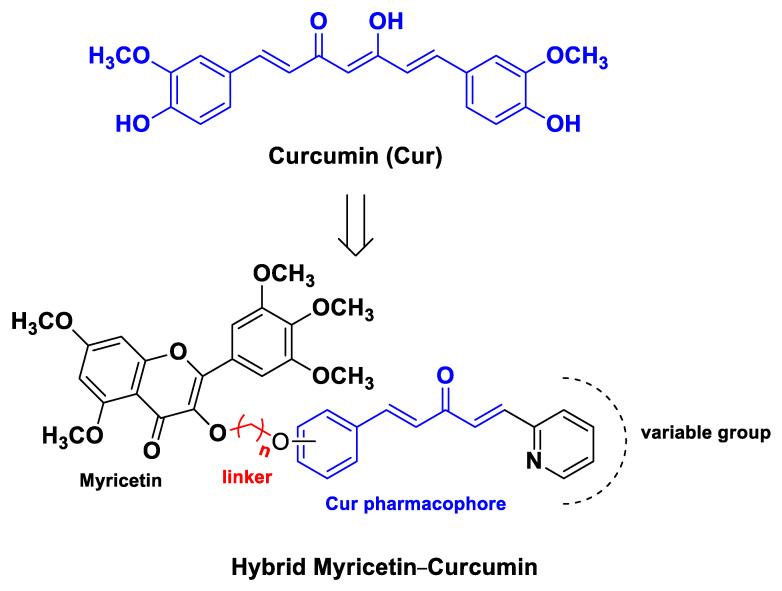
Myricetin–Cur hybrids developed by Wang et al. [63]. For the most active compound, *n* = 3.

A representative and extensive study on the design of hybrid molecules that can be categorized as codrugs was carried out by Wang et al. [63]. The authors synthesized 20 Cur–hybrids containing the flavonoid myricetin or coumarin, two phytochemicals endowed with proven anticancer properties [66,67], as potential anticancer agents targeting the enzyme TrxR, whose overactivity is associated with the development and progression of different types of aggressive cancers [68]. Biological assessments of anticancer activity showed that the myricetin-Cur hybrid compound 5,7-dimethoxy-3-(3-(2-((1*E*,4*E*)-3-oxo-5-(pyridin-2-yl)penta-1,4-dien-1-yl)phenoxy)propoxy)-2-(3,4,5-trimethoxyphenyl)-4*H*-chromen-4-one (Figure 3) is able to induce gastric cancer cell (SGC-7901) apoptosis by the accumulation of ROS, repressing mitochondrial function and inhibiting TrxR activity. Moreover, apoptosis and inhibition of proliferation of gastric cells were reversed in the assays wherein cells underwent preincubation with *N*-acetylcysteine as a result of the decrease in ROS formation and protection of mitochondrial function, underlining the importance of the Trx system for anticancer therapy [63].

**Figure 4:**
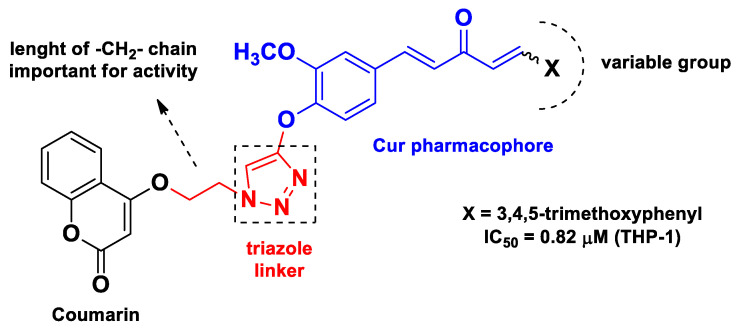
Coumarin–Cur hybrids developed by Bedi’s research group [58].

A similar approach, which entails the use of the monocarbonyl pharmacophoric framework for the construction of the hybrids, was previously undertaken by Bedi’s research group [58]. In this case, the authors successfully obtained coumarin–Cur hybrids by means of a triazole linker generated by a click chemistry reaction between an alkyne moiety introduced at the -OH phenol group of Cur and an azide group appended to the coumarin scaffold. These so-named C_5_-curcuminoid–coumarin hybrids showed significant antiproliferative activity in vitro against different cancerous cell lines, such as the THP-1, COLO-205, and HCT-116 cell lines, whereas the PC-3 cell line was resistant. The most active compounds were selected for further testing as tubulin polymerization inhibitors, and the mechanism was validated by docking studies. The structure of the most active compound and structure–activity relationship (SAR) insights are depicted in Figure 4.

**Figure 5:**
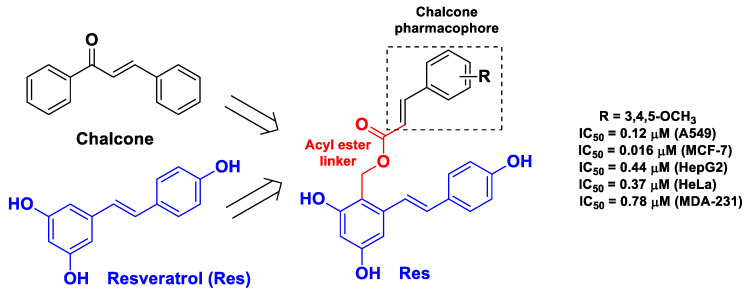
Structure and anticancer activity of the most potent chalcone–Res hybrid developed by Yin et al. [49].

The same research group previously designed and synthesized similar hybrids for which they used the indole derivative isatin as an active component to join to Cur and support the binding with tubulin. The hybrids were evaluated toward a wider panel of cancerous cell lines and were found to be active against the THP-1, COLO-205, HCT-116, and PC-3 cancerous cell lines [59]. In the context of the tubulin polymerization inhibitors as potential anticancer agents, a recent study was carried out by Yin et al. [49]. The design of these hybrids consists of the connection of Res with the pharmacophore of chalcone through a flexible acyl ester group [49]. Chalconoids are known to exert anticancer activity targeting tubulin polymerization [69]. The structure and activity of the most potent compound are presented in Figure 5.

**Figure 6:**
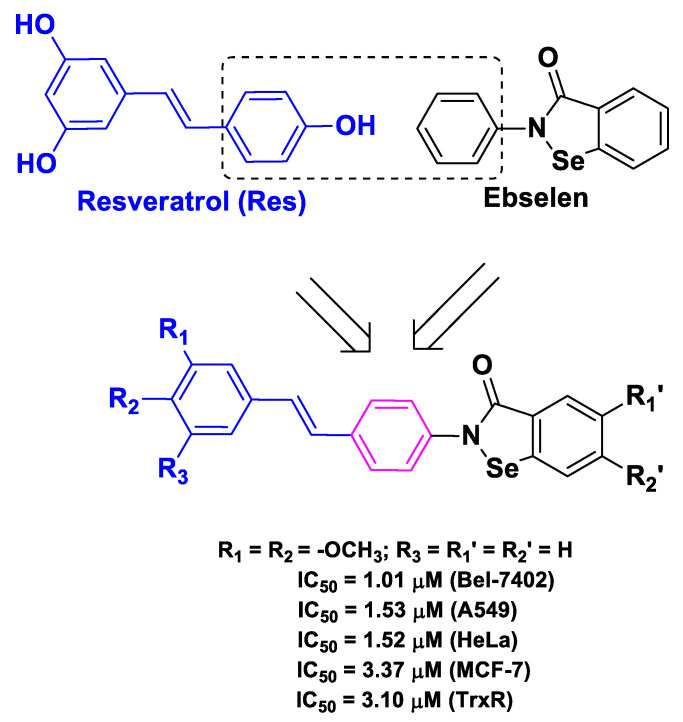
Structure and activity of the most potent chimeric compound developed by Yan et al. [48].

Yan et al. reported an example of the design of chimeric hybrids, wherein they combined in the same chemical entity the pharmacophores of Res and ebselen [48], a synthetic organoselenium derivative (which essentially acts by mimicking glutathione peroxidase) endowed with a broad range of biological activities that can be exploited in anticancer therapy [70]. The resulting benzoselenazole–stilbene hybrids exhibited remarkable antiproliferative activities against four human cancer cell lines, namely Bel-7402, A549, HeLa, and MCF-7. Moreover, it has been demonstrated that these hybrids possess good TrxR inhibitory activity (Figure 6). Cell cycle arrest and apoptosis studies were also performed on Bel-7402 cells, indicating a significant increase in intracellular ROS [48].

**Figure 7:**
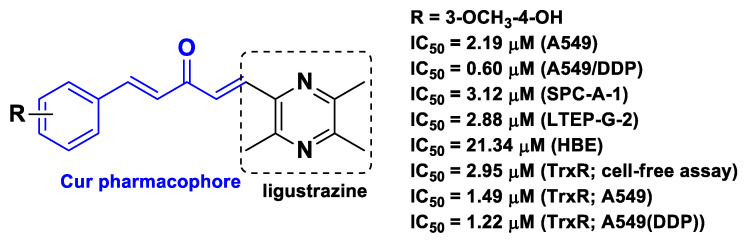
Structure and activity of the most potent chimeric compound developed by Ai et al. [57].

As the strategy to affect the Trx system turns out to be effective for a selective target-based anticancer drug design, another research group aimed to hybrid molecules aimed at this target. Ai et al. obtained chimeric hybrids by merging the pharmacophore of Cur with ligustrazine (tetramethylpyrazine; Figure 7) [57], the bioactive ingredient of a Chinese herb extract used for the treatment of cardiovascular and cerebrovascular diseases [71]. These hybrids significantly inhibited the proliferation of drug-sensitive (A549, SPC-A-1, LTEP-G-2) and drug-resistant (A549/DDP) lung cancer cells with negligible effects on non-tumor lung epithelial-like cells (Human Bronchial Epithelial cells, HBE) (Figure 7). Furthermore, they inhibited the Trx system, promoted intracellular ROS accumulation (as expected because of the presence of the ROS promoting agent ligustrazine), and induced apoptosis (assessed for the most potent derivative). Additional in vitro studies showed also that these hybrids inhibit different tumor-related pathways, and in vivo studies (A549/DDP xenografts) confirmed the antitumor efficacy [57].

**Figure 8:**
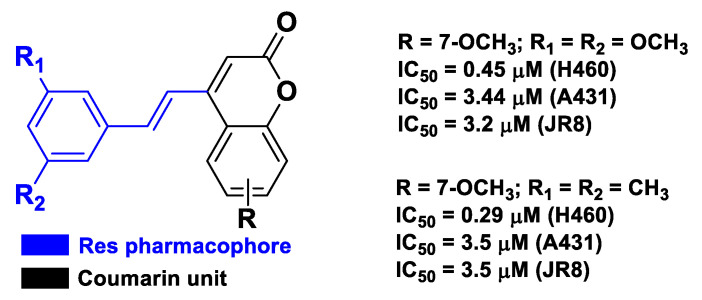
Structure and activity of the two most potent compounds developed by Belluti et al. [56].

Res-based chimeric hybrids were also obtained by combining the pharmacophore Res and the abovementioned coumarin. In this regard, Belluti et al. synthesized stilbene–coumarin derivatives with excellent antiproliferative and proapoptotic activity against a panel of tumor cell lines, namely H460, A431, and JR8 [56]. SAR analysis of this set of compounds indicated that the 7-methoxycoumarin nucleus and the 3,5-disubstitution pattern of the trans-vinylbenzene moiety of Res presented the most promising structural features to achieve excellent antitumor and proapoptotic activity (Figure 8).

**Figure 9:**
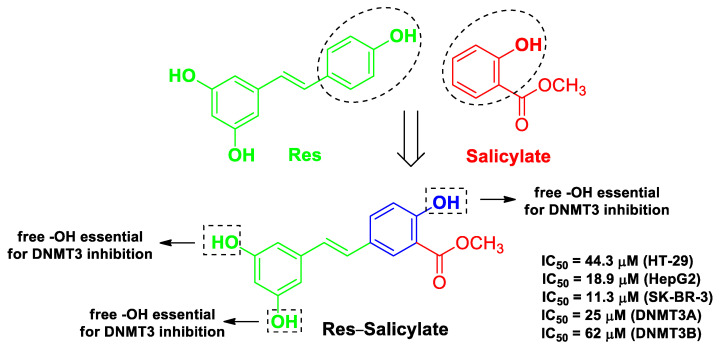
Structure, activity, and SAR insights of the most potent chimeric compound developed by Aldawsari et al. [50].

Aldawsari et al. aimed to develop hybrid molecules by merging the pharmacophores Res and acetylsalicylic acid [50]. The intended targets in this case were DNA methyltransferases (DNMTs), key enzymes involved in carcinogenesis whose expression is decreased by Res [72]. These Res–salicylate hybrids showed considerable cytotoxicity against three human cancer cell lines (HT-29, HepG2, and SK-BR-3; Figure 9) compared with Res, and docking studies indicated selective inhibition of the isoforms DNMT3A and DNMT3B.

On this basis, and in the attempt to improve the bioavailability and reduce the catabolism of this type of hybrid, Salla et al. more recently synthesized Res–aspirin hybrid compounds, which showed antiproliferative and anti-inflammatory activities both in vitro (HCT-116 cells) and in vivo (reduction of the intestinal tumor burden in a xenograft murine model with HCT-116 cells). Additionally, the authors provided details on how these hybrids inhibit the activation of several tumor signaling factors, such as nuclear factor-κB (NF-κB), sirtuin, and AMP-activated protein kinase (AMPK) [55].

**Figure 10:**
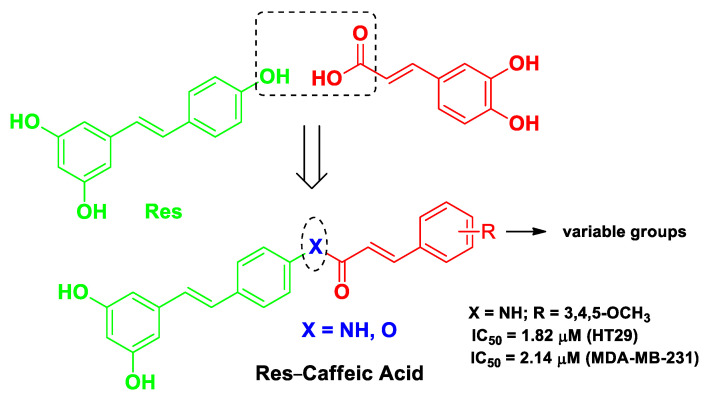
Structure and activity of the most potent hybrid compound developed by Li et al. [51].

Another tumor target of growing interest is STAT3 [73]. Simultaneously disrupting the acetylation and phosphorylation of this cytoplasmic protein was hypothesized to be particularly effective in treating cancers [74]. Li et al. supported this hypothesis for the first time by obtaining two series of Res–caffeic acid hybrids, aiming to regulate both the acetylation (Res component) and phosphorylation (caffeic acid component) of STAT3. The two bioactive molecules were joined by means of a cleavable amide or ester moiety (an example of a fused drug) with the aim of enhancing the bioavailability of the single components. The most potent compound, (*E*)-*N*-(4-(3,5-dimethoxystyryl)phenyl)-3-(3,4,5-trimethoxyphenyl)acrylamide (Figure 10), exhibited remarkable antitumor activity against two cancer cell lines (HT29 and MDA-MB-231) and docking studies confirmed the binding with the intended target. Furthermore, this compound was also effective in vivo in a mouse xenograft model bearing breast cancer 4T1 cells [51].

**Figure 11:**
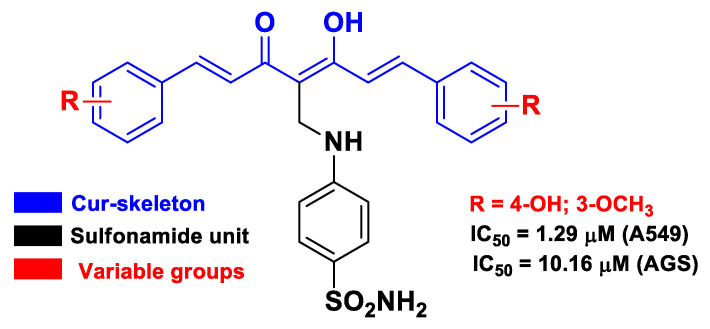
Structure and activity of the most potent hybrid compound developed by Banuppriya et al. [60].

Banuppriya et al. proposed Cur–sulfonamide fused hybrids as inhibitors of the tyrosine kinase domain of EGFR [60], another attractive target for the development of novel anticancer agents [75]. These Cur-based hybrids were obtained by introducing the sulfanilamide unit in the active methylene group of Cur and exhibited antiproliferative activity against two cancer cell lines (AGS and A549) with remarkable antioxidant and anti-inflammatory effects (Figure 11). The molecular docking performed against various EGFR isoforms validated the interaction with the target [60].

**Figure 12:**
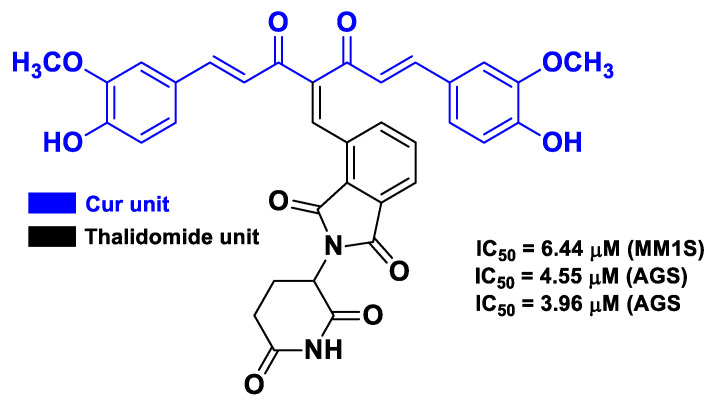
Structure and activity of the most potent hybrid compound developed by Liu et al. [65].

The reactivity of the methylene group between the two carbonyls of Cur was also exploited by Liu et al. to connect thalidomide and Cur in a hybrid compound, which showed in vitro antiproliferative activity against three multiple myeloma cell lines (MM1S, RPMI8226, and U266) and inhibitory effects on NF-κB activation in A549 cells [65] (Figure 12). Further studies also demonstrated that this compound induced apoptosis in U266 cells via ROS production and G1/S cell cycle arrest. The authors also synthesized other Cur–thalidomide hybrids, but the abovementioned fused hybrid was the most effective anticancer agent.

**Figure 13:**
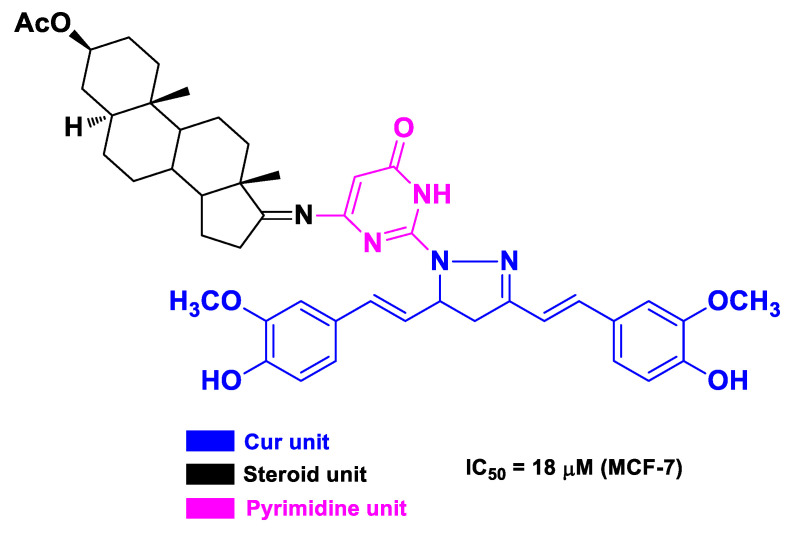
Structure and activity of the hetero-steroid–Cur hybrid developed by Elmegeed’s research group [61].

The reactivity of the β-diketone moiety of Cur was also exploited by Elmegeed’s research group to achieve a hetero-steroid–Cur hybrid as an anti-breast cancer agent [61]. Specifically, the steroid scaffold was first functionalized at C17 with a pyrimidine nucleus bearing a hydrazine moiety, which in turn was condensed with the dicarbonyl fragment of Cur (Figure 13). The resulting hybrid showed in vitro cytotoxic effects toward MCF-7 cells and downregulated the gene expression levels of CCND1, Survivin, BCL-2, CDC2, P21, and P53 [61].

**Figure 14:**
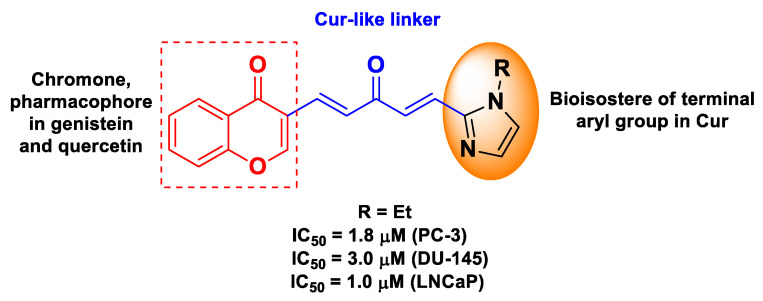
Structure and activity of the most potent hybrid compound developed by Chen et al. [62].

Chen et al. designed Cur-based chimeric hybrids containing the chromone scaffold, a common pharmacophore for natural polyphenols endowed with anticancer properties, such as the isoflavone genistein and the flavonol quercetin [62]. The work was inspired by the synergistic antiproliferative effects of Cur and isoflavones in LNCaP human prostate cancer cells [76,77]. The chromone unit was connected with the Cur-like framework through an aldol condensation reaction, leading to monocarbonyl hybrids wherein the (1*E*,4*E*)-1,4-penta-dien-3-one moiety took the place of the metabolically unstable diketone linker of Cur, and the 1-alkyl-1*H*-imidazol-2-yl group was used as the bioisostere of the terminal aryl group in Cur (Figure 14). For these chimeric hybrids, antiproliferative activity was assessed against three prostate cancer cell lines (PC-3, DU-145, and LNCaP) with remarkable IC_50_ values in the low micromolar range [62].

**Figure 15:**
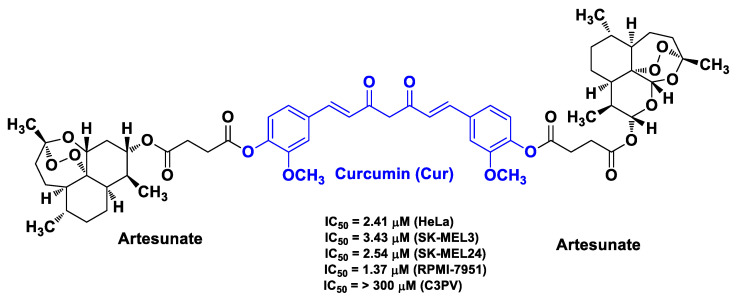
Structure and activity of the most potent dimeric hybrid derivative developed by Botta et al. [64].

Botta et al. synthesized a library of monomeric and dimeric fused hybrids by coupling the semisynthetic derivatives of artemisinin, i.e., artesunate and dihydroartemisinin, with various phytochemicals, i.e., Cur, eugenol, perillyl alcohol, tyrosol, and α- and δ-tocopherol [64]. Artemisinin, as well as its semisynthetic derivatives with improved pharmacological and pharmacokinetic profiles compared with the parent compound, is a sesquiterpene lactone from *Artemisia annua* endowed with remarkable activity against different cancer cell lines exerted by means of the iron-mediated cleavage of its reactive 1,2,4-trioxane ring [78]. Among the hybrids of this series, the dimer artesunate-Cur-artesunate obtained by simple ester linkage between the -OH phenol group of Cur and the -COOH group of artesunate emerged as the most interesting derivative in terms of biological outcomes (Figure 15). Indeed, it exhibited notable antiproliferative (low-micromolar range) activity against HeLa cells and three complementary metastatic melanoma cancer cell lines (SK-MEL3, SK-MEL24, and RPMI-7951) without toxicity against normal cells (human primary fibroblast cell line C3PV). Moreover, assays with the iron-chelating agent deferoxamine surprisingly indicated that this dimeric hybrid does not exert anticancer activity by means of the free radical formation variance exhibited by the other hybrids of this series of derivatives [64].

**Figure 16:**
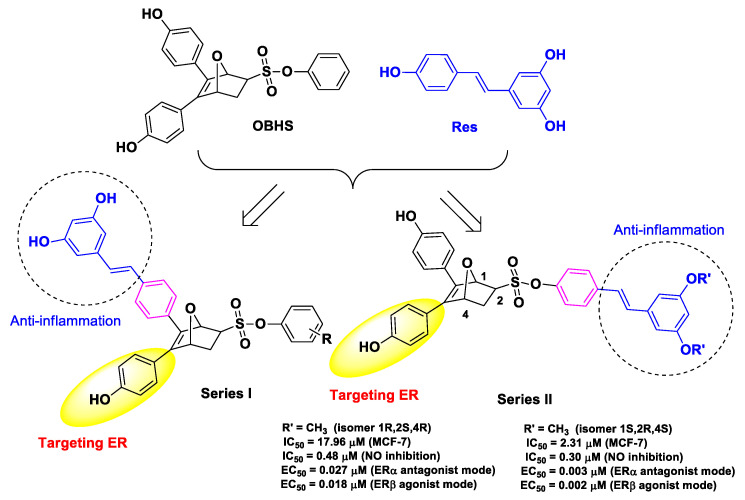
Structure, activity, and SAR insights of the most potent conjugated hybrid derivative developed by Ning et al. [52].

Ning et al. developed dual targeting conjugates by joining Res and oxabicycloheptene sulfonate (OBHS), a structural motif of estrogen receptor (ER), with the aim of suppressing estrogenic and anti-inflammatory activities in breast cancer cells (MCF-7) [52]. The rationale of this design was inspired by evidence that ER/NF-κB interactions are implicated in the progression of breast cancer [79,80]. The authors synthesized two series of OBSH–Res hybrids via the Diels–Alder reaction of furan derivatives and various dienophiles. The most potent compound was found among the type II series and showed remarkable stereospecific binding to ER, excellent NO inhibition in macrophage RAW 264.7 cells, and cytotoxicity in the low micromolar range against MCF-7 cells (Figure 16). In vivo experiments, performed in xenograft models of nude mice injected with MCF-7 cells, showed that this hybrid is more potent than reference tamoxifen [52].

**Figure 17:**
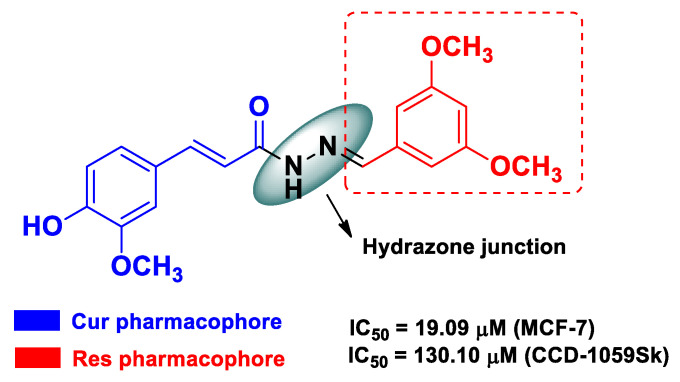
Structure and activity of the most potent Cur–Res hybrid derivative developed by de Freitas Silva et al. [53].

As both Cur and Res possess a plethora of tumor targets, a logical strategy of drug design has been to combine their pharmacophores in one motif in order to modulate the poor ADME (absorption, distribution, metabolism, and excretion) profile of each component and achieve more potent hybrid derivatives. In this context, de Freitas Silva et al. [53] carried out a work in which they joined the two pharmacophores by means of a hydrazone moiety. These Cur–Res hybrids inhibited mitosis progression in estrogen-positive MCF-7 cells inducing G2/M cell cycle arrest and apoptosis without significant toxicity toward normal cells (human normal control fibroblast, CCD-1059Sk) (Figure 17). The anticancer activity of this type of hybrid was also validated in vivo, in which the hydrazone moiety played a role in increasing the coefficient of solubility and absorption of these derivatives [53].

**Figure 18:**
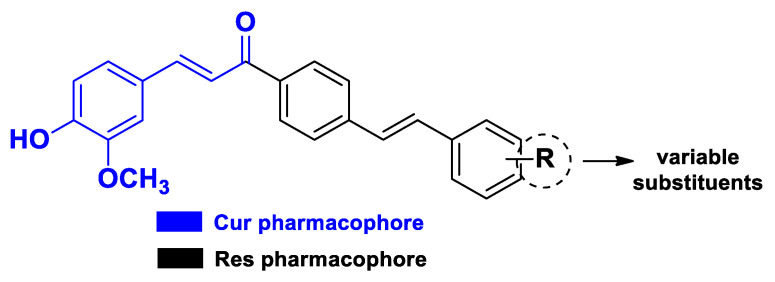
Structure of the Cur–Res hybrid derivatives developed by Hernández et al. [54].

The same type of Cur–Res hybrids without the hydrazone junction were obtained more recently by Hernández et al. in a study wherein they assembled the two pharmacophores of the bioactive molecules by means of cross-coupling reactions between various styrenes and a Br-substituted chalcone (obtained via Claisen–Schmidt condensation) under palladium catalysis (Figure 18). This set of derivatives was evaluated against colorectal cancer cells (SW480 and its metastatic derivative SW620), for which they exhibited IC_50_ values in the micromolar range [54].

## 4. Gold-Based Hybrid Nanosystems

The design of nanomaterials based on metals may be a successful approach to overcome the poor stability and solubility of Cur and Res.

First, a traditional approach is represented by the design of metal–polyphenol complexes. This is easily evident for Cur, which bears an α,β-unsaturated β-diketo moiety with keto–enol isomerization in its chemical structure and thus can readily form a complex with many transition and nontransition metal ions, rare earth ions, and metal oxides, which may help increase its solubility and stability [33]. Metals usually bind to the keto–enol group of Cur by chelation, generally in the ratio 1:1 or 1:2 metal/Cur (although the binding of three Cur molecules has also been reported). One must mention that Cur–metal complexes may affect the biological properties of Cur but may also change those of metals (particularly their toxicity). Metal–Cur complexes are known to have multiple capabilities and have been investigated in cancer applications (copper, zinc, ruthenium, palladium, gallium, nickel, platinum, iron oxide, etc.) [81]. Because of the properties of metals, some of these complexes may be employed not only in cancer treatment but also in diagnosis, and they can improve Cur’s capability to act as a photosensitizer under visible light [82].

However, given the vastness of the topic relating to innovative hybrid materials based on metals and Cur/Res, we chose to limit the discussion to a specific topic that brings together the possibility of pursuing different goals: (1) the design of new hybrid nanosystems for delivery of one or more drugs, which are useful for improving their bioavailability but also actively target a specific tumor cell/tissue and/or respond to the characteristics of the tumor microenvironment; (2) the possibility of exploiting the same system to attack tumor cells not only through chemotherapy but also with other therapeutic strategies, such as photodynamic therapy (PDT); and (3) the preparation of the pharmaceutical system by environmentally friendly (green) routes. As described below in more detail, all these properties can be found in hybrid nanosystems based on gold.

Metallic nanoparticles have been of significant interest in the past two decades as a result of their possible use in the pharmacological treatment of several diseases. Particular attention was devoted to gold nanoparticles (AuNPs) which, because of their unique optical, physical, and surface properties, together with their safety and biocompatibility, are emerging tools for biomedical and pharmaceutical applications, particularly for drug delivery (as drug delivery systems, DDSs), PDT, and photothermal therapy (PTT), as well as for biological optical imaging, and are thus promising candidates for the diagnosis and treatment of different types of cancer [83,84,85,86,87,88,89,90].

AuNPs typically enter cells via endocytosis, and their cellular uptake can be affected by modifying their size, shape, and surface chemistry. Concerning the employment of DDSs, AuNPs can increase the cellular uptake of some drugs and, especially in the case of nanomaterial, are functionalized to target specific cells or tissues (Table 3 and Table 4).

For example, the Cur and lipoic acid (LA) loaded gold–iron oxide nanocomposite developed by Ghorbani et al. [102] has been functionalized with GSH to increase cellular uptake of the system in GSH receptor-positive astrocyte cells, thus ameliorating its ability to bypass the blood–brain barrier. Mahalunkar et al. [105] and Mathew et al. [103] employed folic acid (FA) in Cur-loaded systems projected to target cancer cells that overexpress membrane-associated FA receptors in comparison with normal tissues. Conjugation with the MUC-1 (mucin-1) aptamer has allowed the Cur-loaded system described by Alibolandi et al. [101] to target colorectal adenocarcinoma cells since MUC-1 is overexpressed on several tumor cells and can modulate their invasive and metastatic potential.

In addition, the acidic tumor microenvironment is an important target for the delivery of drugs to a wide variety of malignant tumors. The pH value is more acidic in tumoral tissue (approximately 6.5–6.8) than in normal tissue, and an even lower pH (approximately 5) can be found in tumor cells. The natural polysaccharide chitosan (CTS) has been used by Wang et al. [98] and Rao et al. [107] for the development of AuNP-based DDSs to provide a pH-responsive Cur or Res drug release since its amino groups can be protonated under acidic conditions.

Generally, tumor cells are more thermally sensitive than normal cells because of poor blood supply and irregular vasculature, so moderate hyperthermia can significantly inhibit their growth without damaging healthy cells. As mentioned before, AuNPs are very effective in PTT and PDT. In fact, their tunable surface plasmon resonance (SPR) property allows AuNPs to absorb near-infrared (NIR) light and convert light into heat, further inducing local hyperthermia and cell destruction. It is evident that the employment of AuNPs has several advantages since the treatment can be efficiently targeted to specific tissues and tumors in deep tissues and may be combined with drug therapy using AuNPs as DDSs. Several NIR-responsive AuNP-based nanosystems were developed to deliver Cur or Res to cancer cells for the treatment of malignant melanoma [97,98,108,109,110,111,112,113] and breast and cervical cancer [105,107] (Table 3 and Table 4).

Another alternative for cancer therapy is represented by ultrasound therapy (UST), in which ROS generated by ultrasonic activation of a sonosensitizer are employed to kill cancer cells. Nanomaterials such as AuNPs can work as novel sonosensitizers upon exposure to ultrasound waves. The NIR responsive and sonosensitive system developed by Kayani et al. [110] takes advantage of the sonosensitizer properties of AuNPs as well as of Cur.

**Figure 19:**
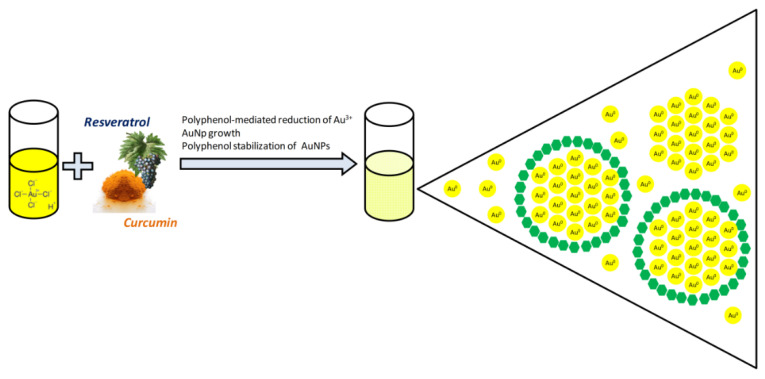
Schematic description of the green synthesis of AuNPs starting from chloroauric acid (HAuCl_4_) and using Cur or Res as reducing and stabilizing agents.

Beyond this, another important factor to consider is that several synthetic approaches have recently been carried out to obtain AuNPs by environmentally friendly (green) routes and economical large-scale methods allowing reduced use of hazardous chemicals and improved material/energy efficiency [19,114,115,116,117]. These eco-friendly synthetic approaches essentially entail the use of phytochemicals (plant extracts or plant-derived compounds) to reduce auric compounds into elemental gold atoms, which bind together to form nanoparticles. The byproducts of the redox reaction or the natural reducing agent itself eventually stabilize the newly formed AuNPs by adsorption on their surfaces [118] (Figure 19).

An application example of a green and cost-effective synthesis of AuNPs functionalized with a natural anticancer agent was reported by Sindhu et al. [119]. To achieve the hybrid system, the authors used Cur both as a reducing agent and a capping agent in a one-step synthesis. The standard procedure for the synthesis AuNPs (Turkevich method) [120] entails the employment of sodium citrate (alone or with the addition of another reducing agent) [121] to afford the reduction Au^3+^ to Au^0^ starting from chloroauric acid (HAuCl_4_) and to stabilize the newly formed AuNPs. Additionally, this method often entails the use of surfactants and, in the case of the preparation of Cur-containing AuNPs (also indicated as Cur@AuNPs), the use of solubilizing agents to favor the incorporation of the bioactive component. However, it is known that citrate-coated AuNPs exert some degree of toxicity attributable to citrate [122,123] and the use of surfactants such as quaternary ammonium salts (e.g., cetyltrimethylammonium bromide (CTAB)) for AuNP synthesis may result in toxic responses by cellular systems [124]. The authors solubilized Cur at an alkaline pH (9.2–9.6) and exploited the reducing properties of the three -OH groups (transformed in two phenolate groups and one enolate group by adding K_2_CO_3_; Cur^3−^) of Cur to achieve in sequence the reduction of HAuCl_4_, the nucleation of the Au^0^ atoms, the growth and cleavage of the clusters, and the maturation of the stabilized form aided by ionic interaction Cur^3−^/Au^3+^ at the surface of the resulting AuNPs. The pH range of 9.2–9.6 played a key role in the achievement of the final Cur@AuNPs hybrid, as at higher pH values, Cur might degrade to condensed products, whereas at lower pH values, it is not efficiently solubilized. This Cur-conjugated AuNPs hybrid system showed good biocompatibility in vitro toward human blood cells (RBCs and PBMN cells) and interesting prospects for application in anticancer therapy [119].

Another noteworthy approach that omits the use of both chemical reducing agents and surfactants to obtain a Cur-conjugated hybrid nanosystem was carried out by Govindaraju et al. [99]. This research group used bovine serum albumin (BSA) and Cur as reducing and stabilizing agents. The synthetic procedure is similar to the one described above; the alkaline medium to solubilize Cur and improve its reducing power was adjusted by adding NaOH, and the presence of BSA promoted the formation of quantum-sized Au nanoclusters during the nucleation and growth process [125]. Since clusters consist of a small number of atoms compared with NPs and exhibit a strong fluorescence under irradiation, this Au-based hybrid nanosystem (particle size of 1–3 nm) is suitable for both bioimaging applications and anticancer therapy. As a matter of fact, the obtained results evidenced that these Cur–Au nanoclusters exhibited a strong red fluorescence and high toxicity in HeLa cancer cells and negligible toxicity toward COS-7 normal kidney cells [99].

Similarly to Cur, other (poly)phenolic phytochemicals can be employed as reducing and capping agents for the green synthesis of AuNPs, leading to hybrid nanoformulations with excellent bioavailability, anticancer activity, and cellular uptake properties [126]. For instance, Park et al. [93] used Res to obtain Res-conjugated AuNPs with a remarkable ability to suppress migration and invasion in breast cancer (MCF-7cells treated with the tumor promoter 12-*O*-tetradecanoylphorbol-13-acetate (TPA) compared with treatment with Res alone. Furthermore, these Res@AuNPs turned out to be effective in downregulating the enzymatic activity and/or expression of a panel of signaling targets involved in tumor progression and metastasis, such as MMP-9, cyclooxygenase-2 (COX-2), NF-κB, AP-1 (activator protein-1), PI3K/Akt (phosphatidylinositol 3-kinase/protein kinase B), and ERK, as well as in upregulating heme oxygenase-1 (HO-1) expression [93].

Wang et al. [98] proposed a surfactant-free synthesis of Res-conjugated hollow AuNPs for anticancer theranostic applications by exploiting the unique plasmonic properties of these Au-based nanocomposites and the synergistic effects of AuNPs and Res in this type of application [127]. The authors prepared these multifunctional nanocomposites through the galvanic replacement reaction between HAuCl_4_ and preformed sacrificial citrate-stabilized Ag seeds in the presence of Res as a reducing and coating agent. The obtained Res@Au hollow NPs showed high photothermal performance in NIR (specifically after 808 nm laser irradiation), good stability, biocompatibility, and a notable cytotoxicity profile in malignant melanoma cancer cells (A375) [98]. Comparable outcomes in terms of photothermal responsivity were obtained by Rahimi-Moghaddam et al. [112], who employed the Cur@AuNPs hybrid system with an average hydrodynamic particle size of 25.8 nm as a photo–thermo conversion agent using diode lasers emitting at 808 nm and 650 nm as light sources. Their hybrid system proved to be much more efficacious in vitro against 4T1 breast cancer cells after laser irradiation at 808 nm than after irradiation at 650 nm [112]. Previously, the same research group used a refined version of this hybrid system for the PTT of melanoma tumors [111]. Specifically, they employed PEG as a coating agent for the Cur@AuNPs because of its ability to improve biocompatibility, stability, blood retention time, and tumor tissue accumulation of the delivery system, along with its ability to positively affect the surface plasmon resonance properties of the AuNPs (e.g., shift to NIR wavelengths, particularly suitable in PTT) [128,129]. Upon laser irradiation at 808 nm, PEG–Cur@AuNPs showed in vitro efficacy against mouse malignant melanoma cell lines C540 (B16/F10) and in vivo efficacy against implanted melanoma tumors in C57/inbred mice [111]. More recently, Kayani et al. [110] optimized their own PEG–Cur@AuNPs hybrid system for both the PTT and sonodynamic treatment (SDT) of melanoma cancer, whose in vitro and in vivo efficacy was assessed using the same melanoma cell lines and animal models. New PEG–Cur@AuNPs caused localized hyperthermia and apoptosis of tumor cells upon PTT (laser irradiation 808 nm) and ROS production upon SDT (ultrasound exposure at 1.0 MHz) with tangible synergistic effects [110].

In order to increase the corona of Res on AuNPs (and thus the therapeutic efficacy of the Res@AuNPs hybrid system), Thipe et al. carried out a green nanotechnology synthetic approach in which they used gum arabic as an encapsulating agent to increase the conjugation of Res onto the surface of AuNPs and the overall stability of the resulting hybrid system [94]. Gum arabic, being a complex polysaccharide with a highly branched structure, provided an ideal support matrix for the loading and delivery of Res. This enriched hybrid system was obtained by the simple addition of NaAuCl_4_ (in place of the classic HAuCl_4_) to an aqueous solution of Res (the reducing and capping agent) and gum arabic under vigorous stirring, and provided superior in vitro anti-tumor activity against human breast (MDAMB-231), pancreatic (PANC-1), and prostate (PC-3) cancer cells with respect to the parent Res@AuNPs system [94]. The therapeutic potential of the Res@AuNPs system can be considerably increased by adding the non-ionic (and relatively non-toxic) surfactant Tween-20 as a loading enhancer, as was recently demonstrated by Fadel et al. [91]. Tween-20 binds to the hydrophilic surface of the AuNPs, then undergoes self-assembly in a bilayer coating structure that functions as a reservoir for Res. The cytotoxicity of these Res-loaded Au-based hybrid nanosystems toward HepG2 cell lines turned out to be ~nine times more potent compared with free Res at the same concentration [91]. The large anti-hepatoma effects of the Res@AuNPs compared to Res alone were further confirmed by Zhang et al., who enabled the transfer of these exceptional outcomes in vivo by means of xenograft models of nude mice injected with HepG2 cells [92]. These xenografts studies evidenced that Res@AuNPs inhibit tumor growth, promote apoptosis, and decrease the expression of VEGF in tumor tissue without substantially affecting (as determined by hematoxylin and eosin staining) vital organs such as the liver, heart, spleen, and kidney. The in vitro antiproliferative activity of Res@AuNPs (synthesized without the use of surfactants) was ~6.5 times higher compared with Res (IC_50_ = 3.84 µg/mL vs. IC_50_ = 24.74 µg/mL in HepG2 cells), with negligible toxicity toward healthy cells (L02). Additionally, the authors demonstrated that Res@AuNPs upregulate the expression of the proapoptotic regulators caspase-8 and Bax and downregulate the expression of procaspase-3, procaspase-9, and the PI3K/Akt survival signaling pathways both in HepG2 cells and a xenograft model [92].

A topical trend of nanomedicine is that of conjugating AuNPs with the antitumor drug doxorubicin (Dox) to enhance its bioavailability and limit its side effects. Dox self-assembles in an aqueous medium or PBS on the surface of AuNPs (colloidal solution) by means of supramolecular interactions [130]. On the other hand, Res is known to exert synergistic anticancer activity with Dox, reduce its toxicity, and reverse its multidrug resistance in different cancerous cell lines [131,132,133]. In this regard, Tomoaia et al. prepared nanocomplexes of Dox and Res@AuNPs, which were highly effective in vitro against two human cervical cancer cell lines, namely HeLa (HPV-18 positive) and CaSki (HPV-16) cells. The authors also proved that the Dox effects on cell viability were mediated by both Dox@AuNPs and Dox-Res@AuNPs through an apoptotic mechanism [96]. Similar results were obtained earlier by Mohanty et al. in research (which was likely inspired by the Tomoaia group’s work) in which they obtained Res@AuNPs with high stability in various aqueous media mimicking physiological conditions and biocompatibility (assessed in fibroblast cells by MTT assay). These Res@AuNPs were eventually loaded with Dox by the simple addition of the latter to the hybrid system and sonication, and the resulting nanocomplexes Dox-Res@AuNPs were evaluated for their in vitro anticancer activity against a human glioma carcinoma cell line (LN 229), showing clear efficacy [95].

The drug delivery and therapeutic potential of AuNPs can be greatly enhanced by joining to these platforms another excellent drug delivery vehicle, liposomes (Lips) [134,135,136], which can be triggered by various chemical/physical stimuli [137,138]. The resulting hybrid systems are multifunctional, as both AuNPs and Lips can be widely decorated on their surface to achieve active/passive targeting delivery systems and/or stimuli-responsive systems. An explanatory example of this is the multifunctional platform developed by Wang et al. [97]. Starting from Res-loaded Lips, the authors first coated these amphipathic and sphere-shaped vesicles using CTS, a positively charged and pH-responsive polysaccharide that can adsorb on the negatively charged surface of Lips by electrostatic interactions and reverse their surface charge [139]. CTS@Res–Lips were then added to Au seeds (negatively charged) and incubated to present AuNPs on the surface of Lips (CTS@Res-Lips@AuNPs). The AuNPs underwent nanoshell formation (AuNSs) by means of a method known as the seed-growth method, which entails the use of AuCl_3_ (the growth component) and ascorbic acid (the reducing agent). The final hybrid system (CTS@Res–Lips@AuNSs) was thermo-pH dual responsive (on-demand drug release) and suitable for chemotherapy and PTT (synergistic effect) assessed in Hela cells [97].

**Figure 20:**
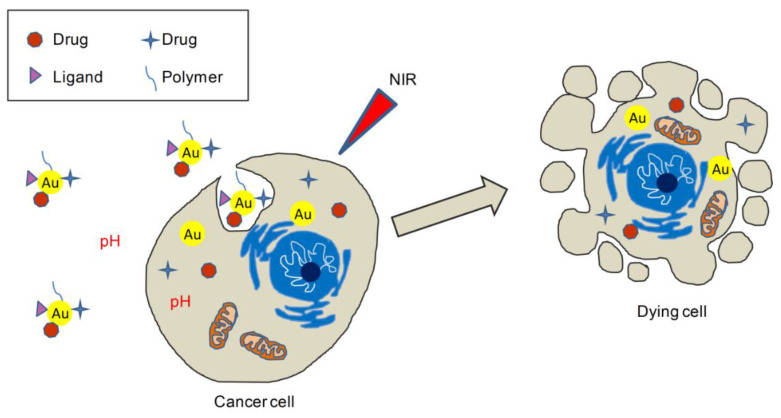
AuNPs may be employed as active targeted, NIR- and pH-responsive drug delivery systems, allowing a synergic effect between drug chemotherapy and PTT/PTD in cancer cells.

Singh et al. reported the synthesis of Cur–Lips@AuNPs as in situ adjuvant for PTT of skin cancer assessed in B16/F10 melanoma cell lines [109]. The authors employed Lips made up of hydrogenated soya phosphatidyl choline (HSPC) (which shows superior targeting properties compared with other classic phospholipids) [140] to load the bioactive molecule. Then, Cur–Lips were coated with AuNPs by the simple and sequential addition of HAuCl_4_ and ascorbic acid. The latter compound, a weak reducing agent, determines the change of the surface charge of Lips because of the partial conversion of Au^3+^ to Au^1+^, leading to enhanced stability and intracellular uptake of the final hybrid system. Cur–Lips@AuNPs showed photothermal efficacy and triggered the release of Cur in NIR (780 nm) [109]. The same research group elaborated this system and implemented it in in vivo PTT studies performed in a murine model (C57BL/6 female mice) of induced melanoma (B16 cells), obtaining a depot version of the drug delivery platform with sustained and prolonged (>10 days) release of Cur in situ upon laser irradiation. This is because of the occurrence of an in situ crystalline state transition of Cur, from nano (within Lips) to micro (release of Cur from Lips and in situ coalescence of the nanocrystals), upon photothermal triggering. The in vitro studies confirmed also the requirement of the adjuvant (i.e., Cur) for an efficient therapeutic coverage (>1.5-fold) [108] (Figure 20).

Since cancerous cells overexpress specific receptors on their surfaces [141], another widely exploited strategy of nanomedicine to further functionalize the extraordinary Au-based hybrid delivery platforms discussed so far with appropriate ligands to achieve systems for targeted therapy and/or diagnostics. FA, which has two free -COOH groups and is a tumor-targeting ligand, is particularly suited for this type of functionalization [142]. Mathew et al. combined the targeting properties of FA and the excellent optical and delivery properties of Au quantum clusters (AuQCs) in a Cur-containing multifunctional hybrid system for anticancer therapy and bioimaging [103]. First, Au clusters of an average size of ~2 nm were coated with gliadin, a class of Pro- and Gln-rich proteins present in the wheat gluten with a high capability of binding hydrophobic molecules also due to the high content of nonpolar amino acids in its primary structure [143]. Then, AuQCs@gliadin was loaded with Cur, and the resulting hybrid AuQCs@gliadin@Cur was functionalized with FA and conjugated with additional Cur by coupling reaction (EDC/NHS). The final hybrid system AuQCs@gliadin–FA–Cur showed an encapsulation efficiency of Cur of 98%, pH-stability (up to pH = 8.5), and pH-sensitivity (sustained and prolonged release of Cur up to 60 h). Moreover, it was effective in vitro against breast cancer cells (MDA-MB239) with the lowest toxicity against normal L929 cells (as they also express FA receptors). Cellular uptake studies also showed high efficacy toward cancerous cell lines (MDA-MB239 and C6 glioma cells) with respect to normal L929-normal cells [103].

Mahalunkar et al. obtained a comparable tumor-targeting and pH-responsive (80% drug release at a pH of 5.3) system by using polyvinylpyrrolidone (PVP) as a coating agent for AuNPs [105]. PVP forms a thin layer around the AuNPs that can be activated to produce free -NH_2_ groups on the surface. Activated AuNPs@PVP undergo conjugation in sequence with Cur and FA (in turn separately activated at the -COOH groups with EDC/NHS) by standard coupling chemistry. The resulting hybrid system AuNPs@PVP–Cur–FA showed remarkable antiproliferative activity in vitro against different types of breast cancer cell lines, namely human breast adenocarcinoma MDA-MB231 and MCF-7 cells and mouse mammary carcinoma 4T1 cells, without harming normal cell lines (L929 and MCF 10A). The antiproliferative activity was more pronounced toward estrogen/progesterone receptor (ER/PR)-negative cells (MDA-MB231 and 4T1) than toward ER/PR-positive cells (MCF-7). The anticancer efficacy of this hybrid system was also assessed in vivo by means of a breast cancer orthotopic mouse model (tumors induced by 4T1 cells in Balb/c female mice) [105].

Inorganic–organic hybrid materials that can self-assemble into defined multifunctional templates are also topical in the development of drug delivery nanosystems [144]. In regard to AuNPs, a strategy often undertaken is to combine them with proteins because of the fine characteristics of the latter. Dai et al. proposed hybrid templates composed of protein diblock copolymers, namely EC and CE (E: Elastin-like peptide; C: Cartilage oligomeric matrix protein), and AuNPs. The two diblock copolymers EC and CE were each genetically engineered with a His_6_-tag at an *N*-terminus to allow the in situ synthesis of these hybrid biomaterials via AuNPs template with the aid of the reducing agent NaBH_4_. The hybrid nanocomposites were eventually loaded with Cur to afford systems with increased drug loading capacity (> sevenfold), slower drug release profile (~50%), and enhanced cellular uptake of Cur (>twofold) compared with protein polymers alone, as assessed in MCF-7 cells [104]. Alibolandi et al. developed multifunctional inorganic–organic hybrid platforms for theranostic applications in anticancer therapy by encapsulating AuNPs and Cur in poly(amidoamine) (PAMAM) dendrimers [101], spherical and highly branched macromolecules with an extraordinary capacity of loading hydrophobic molecules and stabilized metal NPs. PAMAM first underwent PEGylation (EDC/NHS coupling chemistry) to further enhance the drug loading capacity of organic polymer; the PEG–PAMAM conjugate was then added to HAuCl_4_ and NaBH_4_ (one-pot reduction chemistry as described above), and the resulting hybrid PEG–PAMAM–AuNPs were eventually loaded with Cur. Additionally, to impart tumor-targeting properties to the hybrid platforms, the authors conjugated MUC-1 DNA aptamer to them via a thiolate–maleimide (aptamer–PEG) reaction. The in vitro and in vivo biological assessments proved that these Cur-containing multifunctional hybrid systems accumulate in two colon adenocarcinoma cell lines (human HT29 and mouse C26 cells) and may serve as probes for CT imaging and versatile vehicles for anticancer therapy (C26 tumor-bearing mice) [101].

Inorganic materials may also serve on their own as structural templates for substantial upload of AuNPs and bioactive molecules to afford nanocomposites that combine different moieties, producing hybrid systems for the anticancer therapy with superior features and enhanced performance. In this regard, graphene (G) is one of the most renowned materials because of its high planar surface area (suitable for a high rate of functionalization and drug loading), hydrophobicity, and safe profile of G-cell interaction and cellular uptake (particularly required in the design of passive targeting drug delivery platforms) [145,146]. However, the use of G as a raw material presents some limitations, e.g., excessive hydrophobicity and self-aggregation, which can be overcome by the functionalization of G into graphene oxide (GO), which in turn may be further and more easily functionalized and/or hybridized [147,148]. Al-Ani et al. developed a GO-based hybrid nanocomposite containing AuNPs and Cur by a green one-pot synthetic method [100]. The -COOH groups of GO ensured the initial ionic interactions with Au^3+^ (from HAuCl_4_) on the surface of the material; the subsequent addition of alkaline Cur provided the concurrent reduction of the auric salt (Au^3+^ to Au^1+^ and then AuNPs) and GO (GO to rGO) as well as the stabilization of the newly formed hybrid system. The reduction of GO to rGO is an important step to avoid uncontrolled oxidative stress and cellular damaging effects by GO [146,149]. The rGO@AuNPs@Cur hybrid nanocomposites showed high antioxidant and anticancer efficacy in vitro against two human colon cancer cell lines (HT-29 and SW-948), coupled with a high biocompatibility profile assessed in normal human colon (CCD-841) and liver (WRL-68) cells [100]. A similar GO-based hybrid nanocomposite, obtained in a two-step synthesis with the use of a CTS to achieve first the GO-Au nanocomposite using a surfactant (Tween-80) to later enhance the loading of Cur, was obtained by Ramazani et al. [150]. The authors provided a hybrid nanocomposite with notable cancer-specific toxicity evaluated in vitro against breast cancer cells (MCF-7), with no detectable toxicity toward normal healthy cells*,* namely HEK293 and RBC (hemolysis assay), or brine shrimp (*Artemia salina* nauplii) larva [150]. Another outstanding GO-based hybrid nanocomposite with remarkable anticancer activity in vitro against breast cancer cells (MCF-7) and carcinoma lung cells (A549) was developed by Malekmohammadi et. al. [106]. This smart hybrid system consists of a substrate of rGO nanosheets which underwent functionalization via an oil-in-water stratification method with dendritic mesoporous silica (dMS) nanosheets using a surfactant (cetylpyridinium bromide (CPB)), a silica source (tetraethylorthosilicate (TEOS)), and a catalyst (triethylamine (TEA)), to produce the sandwich-like hybrid platforms rGO@dMS. The latter were in turn functionalized by 3-(trimethoxysilylpropyl) diethylenetriamine (Si-DETA) at reflux in order to achieve free -NH_2_ groups on the surface (rGO@dAMS), which allowed conjugation with the active targeting agent FA by simple coupling chemistry (DCC/NHS). The intermediate nanocomposites rGO@dAMS-FA were eventually loaded with Cur and doped with AuNPs, which were separately prepared by a method known as pulsed-laser ablation of an Au plate in water [151]. The final nanocomposite rGO@dAMS–FA@AuNPs–Cur showed a sustained release of Cur at a low pH under NIR laser irradiation and enhanced tumor-specific cellular uptake, thereby acting as an extraordinary stimuli-responsive and targeting platform [106].

Iron oxide (Fe_3_O_4_) NPs also represent a valid inorganic substrate to build up DDSs for theranostic applications in anticancer therapy. When combined with AuNPs, the resulting nanocomposites usually possess more promising properties in terms of biocompatibility and therapeutic efficacy compared with the pristine nanosystems [152]. A representative study in this respect was carried out by Ghorbani et al. [102]. The authors developed a multifunctional hybrid platform based on Fe_3_O_4_@Au NPs as a targeted and stimuli-responsive DDS for brain cancer theranostics using GSH as a targeting agent and Cur as a bioactive component. Fe_3_O_4_ NPs were obtained by a standard method and functionalized at their surface with (3-aminopropyl) triethoxysilane (APTES) to present free -NH_2_ groups (Fe_3_O_4_-NH_2_). AuNPs were obtained by classic procedures (citrate method plus NaBH_4_) as well. Then, Fe_3_O_4_-NH_2_ NPs and AuNPs were combined to obtain the nanocomposite, with Fe_3_O_4_ as the central core and the citrate-stabilized AuNPs as the coating layer. Then, the hybrid Fe_3_O_4_@Au NPs underwent simultaneous conjugation with GSH and Cur through ligand exchange of the thiol groups with citrate of AuNPs. To achieve this, Cur was first conjugated with LA, another bioactive component endowed with antioxidant and neuroprotective properties [153], bearing a disulfide functional group. The efficacy of the Fe_3_O_4_@AuNPs–GSH–LA–Cur nanocomposites was evaluated against cancerous and normal astrocytes (U87MG) showing an approximately twofold increased cellular uptake in GSH receptor-positive astrocytes, an approximately ninefold lower IC_50_ values compared with Cur treatment, high drug loading capacity (~70%) and release, and applicability as a negative magnetic resonance imaging contrast agent [102].

Rao et al. proposed halloysite nanotubes (HNTs) as a delivery platform for the development of Au-based hybrid systems containing Cur for the treatment of breast cancer [107]. HNTs are naturally occurring and biocompatible aluminosilicates with a hollow tubular structure that ensures a high surface of functionalization and loading capacity [154]. The hybrid systems were prepared using a biofriendly method that avoided the employment of reducing agents. HNTS were simply dispersed in water and supplemented with Cur and HAuCl_4_ to promote the in situ formation of AuNPs (hexagonal and rod-shaped structures) loaded with Cur both in the lumen and the surface cage of HNTs. The hybrid systems were eventually coated with bio-adhesive CTS as a polysaccharide. The HNTs@AuNPs@Cur/CTS hybrids showed notable anticancer activity in vitro against MCF-7 cells (IC_50_ = 14.1 μM), much higher than that of Cur (IC_50_ = 47.8 μM). Moreover, they were stimuli-responsive under an acidic environment (with greater Cur release at pH = 5.5) and NIR irradiation, making them suitable for both targeted delivery of drugs and NIR-imaging [107].

A unique, multifunctional, water-dispersible smart hybrid nanosystem was developed by Wu et al. [113]. It consists of Ag/Au bimetallic NPs with a core-shell structure in which the NPs were coated with a gel layer polystyrene (PS; inner shell) to enhance the loading capacity of hydrophobic drugs as Cur and subsequently coated with a gel layer of hydrophilic nonlinear PEG (outer shell) to trigger the release of the pre-loaded Cur after internal (temperature) or external (NIR) stimuli and enhance stability in aqueous media and biocompatibility. The synthetic strategy to achieve these systems first entails obtaining citrate-stabilized AgNPs by a standard reduction method (NaBH_4_); then, the sequential coating of AgNPs with PS (via radical polymerization of styrene, divinylbenzene, and radical initiator AAPH), and PEG (via cross-linking chemistry of the PEG-precursor MEO_2_MA-co-MEO_5_MA and the linker PEGDMA) to produce Ag@PS–PEG hybrid nanogels, which were converted into Ag/Au@PS–PEG hybrid nanogels by galvanic replacement reaction between Ag and Au(III) (from HAuCl_4_). Finally, Cur was loaded to these hybrid nanogels by the complexation method, and the resulting systems were evaluated for their in vitro anticancer activity against B16F10 mouse melanoma cells, showing good drug release and responsivity to NIR. Therefore, these hybrids might be particularly suitable for combined chemo–photothermal treatment compared with chemothermal or photothermal treatment alone [113].

## 5. Perspectives and Conclusions

In recent years, there has been growing interest in the development of hybrid products based on plant-derived bioactive polyphenols, which are useful for the treatment of human cancer. Cur and Res are gaining considerable attention because of their great therapeutic potential, specifically their capability to act as multifunctional antitumoral agents, but their use as drugs is still hampered by their poor bioavailability due to low water solubility, stability, and bioavailability.

Taking this into account, this review systematically described two hybrid strategies (hybrid molecules and hybrid AuNPs), which seem to be among the most promising strategies to overcome the limitations of these polyphenols and at the same time offer additional advantages. In fact, because of the extraordinary variability of the chemical composition and the technologies employed, there is a possibility to obtain Cur- and Res-compounds able to interact simultaneously with more than one cellular target, and this is particularly important for the treatment of complex multigenic and multifactorial diseases such as cancer. On the other hand, there is increased development of Cur- and Res-loaded nanosystems targeted at specific tissues/cells and responsive to particular environmental conditions, such as pH, and external stimuli, such as NIR, thus avoiding damage to healthy cells and obtaining a synergic effect between chemotherapy and PTT/PTD, which is useful in limiting the toxicity resulting from high dose chemotherapy. The present review displayed the most recent innovative research on the design of Cur- and Res-based hyd=brid products as potential candidates for employment in oncology.

These results are very promising, highlighting that rationally designed hybrid products based on plant polyphenols may have extraordinary potential in the treatment of cancer (as well as of other multifactorial diseases), both used alone or in combination with traditional therapeutic approaches. On this basis, we can suppose that the research on hybrid nanoproducts based on Cur and Res, as well as other natural polyphenols, will continue to increase in the coming years, especially for combined therapies.

However, it is also evident that the road that leads to the translation of these data from the laboratory to the clinic is still very long.

First, one must point out that the efficacy of all the hybrid nanoproducts reported in this review was demonstrated only in vitro; just a few of these studies were carried out in vivo, but only in experimental animals. Thus, great caution should be taken in extrapolating this experimental evidence from the cancer cell lines to complex biological systems and then to humans, and further accurate studies are needed to understand the action mechanisms of the innovative products and to verify their potential clinical employment.

Another aspect on which it is necessary to focus is that of safety and biocompatibility. Cur and Res, as well as other natural polyphenols, are generally recognized as safe for humans. However, they might have side effects if introduced at particularly high doses or through formulations able to increase their bioavailability [155,156]. The problem of their possible side effects might be reduced by using them as a component of new hybrid compounds or, especially, in new active targeted delivery systems since this may allow therapeutic effects to be reached at lower doses. On the other hand, it is also true that when bioavailability is modified, the risk/benefit ratio has to be carefully re-evaluated to avoid toxicity related to the drug and/or the entire delivery system. In particular, hybrid compounds are new entities, with pharmacodynamic/pharmacokinetic features different from the drugs on which they are based, and thus not only their efficiency but also their safety must be accurately investigated.

Finally, the increased intricacy of methodologies required to obtain the described hybrid products can also introduce challenges in reproducibility, scale-up/out, and quality control, as well as a significant rise in their cost that can be justified only by producing clinically significant improvements.

In conclusion, hybrid products based on Cur and Res, and more generally on plant-derived polyphenols, offer great promise for the development of new therapeutic approaches for cancer treatment, but further accurate studies are needed to demonstrate their efficacy and safety as well as to optimize their formulation and process.

## Figures and Tables

**Table 1:** Hybrid molecules derived from Res and designed for potential employment in human cancer.

Pharmacophore 1	Pharmacophore 2	Hybrids	Pathology	In Vitro Cell-Free or In Silico Model	In Vitro Cell-Based Model	In Vivo Model	Ref.
Res	ebselen	Benzoselenazole–stilbene hybrids	cancer	inhibition of TrxR	human liver carcinoma Bel-7402		[48]
Res	cinnamic acid	Res linked to cinnamic acid through an acyl ester group	cancer	tubulin polymerization assay; molecular docking with tubulin	lung cancer A549, MCF-7, hepatoma HepG2, cervical cancer HeLa, and breast cancer MDA-231 cells		[49]
Res	salicylate	addition of a carboxylic acid or its methyl ester attached *ortho* to one of the phenol groups present in hydroxystilbene	cancer	molecular docking with human DNMT	human colorectal adenocarcinoma HT-29, hepatoma HepG2 cells, and mammary gland/breast SK-BR-3 cells		[50]
Res	caffeic acid	Res-caffeic acid hybrids possessing an amide linker or an ester linkage	breast cancer	molecular docking with STAT3 protein	human breast cancer MDA-MB-231 cells and colonic carcinoma HT29 cells	female Kunming mice bearing breast cancer 4T1 cells	[51]
Res	obhs	conjugation of Res with OBHS	breast cancer	estrogen receptor ERα antagonistic activity	human breast cancer MCF-7 cells	female Balb/c nude mice inoculated with MCF-7 breast cancer cells	[52]
Res	Cur	an *o*-substituted conjugated-phenyl system from Res linked to a 3-methoxy-4-hydroxycynamoil subunit, with a hydrazone functionality as a spacer	breast cancer		estrogen-positive human breast cancer MCF-7 cells		[53]
Res	Cur	Res linked to a 3-methoxy-4-hydroxycynamoil subunit from Cur	colonic cancer		human colon adenocarcinoma SW480 and SW620 cells		[54]
Res	Aspirin	addition of a carboxylic acid group adjacently to one of the phenols in the Res structure	colonic cancer and intestinal inflammation		normal mouse intestinal ModeK cells and human colon cancer HCT116 cells	C57BL/6 mice bearing HCT116 colon cancer cells; DSS-induced colitis in male C57BL/6 mice	[55]
Res	coumarin	a substituted trans-vinylbenzene moiety on a coumarin backbone	cancer		human lung carcinoma H460, squamous carcinoma A431, and melanoma JR8 cells		[56]

Res: resveratrol; Cur: curcumin; OBHS: oxabicycloheptene sulfonate; TrxR: thioredoxin reductase; DNMT: DNA methyltransferase; STAT3 protein: signal transducer and activator of transcription 3 protein; DSS: dextran sodium sulfate.

**Table 2:** Hybrid molecules derived from Cur and designed for potential employment in human cancer.

Pharmacophore 1	Pharmacophore 2	Hybrids	Pathology	In Vitro Cell-Free or In Silico Model	In Vitro Cell-Based Model	In Vivo Model	Ref.
Cur	ligustrazine	substituting one of the two aromatic rings of Cur analogs with ligustrazine	lung cancer	inhibition of TrxR	human lung cancer A549, drug-resistant human lung cancer A549/DDP cells	athymic BALB/c nude mice inoculated with A549/DDP cells	[57]
Cur	coumarin	monocarbonyl Cur linked to coumarin with a trizole as spacer	cancer	molecular docking with tubulin	human leukemia THP-1, colon adenocarcinoma COLO-205, colorectal cancer HCT-116 cells		[58]
Cur	Isatin	monocarbonyl Cur linked to isatin with a trizole as a spacer	cancer	tubulin polymerization assay; molecular docking with tubulin	human leukemia THP-1, colon adenocarcinoma COLO-205, colorectal cancer HCT-116, prostate cancer PC-3 cells		[59]
Cur	sulfonamide	introduction of sulfanilamide unit into the methylene part of Cur	cancer	molecular docking with EGFR TK	human gastric adenocarcinoma AGS and lung cancer A549 cells		[60]
Cur	steroids	pyrazolocurcumin-pyrimidinyl androstane derivative	breast cancer		human breast cancer MCF-7 cells		[61]
Cur	quercetin or genistein	ester of (1*E*,4*E*)-1,4-penta-dien-3-one (from Cur) and chromone (from quercetin or genistein)	prostate cancer		human prostate cancer androgen-independent PC-3b and DU-145 cells, and androgen-dependent LNCaP cells		[62]
Cur	myricetin	monocarbonyl analogs of curcumin linked to myricetin	gastric cancer		human gastric cancer SGC-7901 cells		[63]
Cur	artesunate	linkage of Cur with two artesunate molecules	melanoma		melanoma SK-MEL3, SK-MEL24, and RPMI-7951 cells		[64]
Cur	thalidomide	thalidomide linked at the methylene position between the two carbonyls of Cur; monoketone Cur linked to thalidomide	multiple myeloma		human multiple myeloma MM1S, RPMI8226, U266 cells		[65]

Cur: curcumin; TrxR: thioredoxin reductase; EGFR TK: Epidermal Growth Factor Receptor tyrosine kina.

**Table 3:** Hybrid systems based on AuNPs and Res and projected as potential candidates for the treatment of human cancer.

Cytotoxic Drug	Hybrid Materials	Aim	Functionalization	Pathology	In Vitro Model	In Vivo Model	Ref.
Res	Res-loaded Au nanospheres	DD		liver cancer	Human liver HepG2 cells		[91]
Res	Res-loaded AuNPs	DD		liver cancer	Human liver HepG2 cells	15 BALC/c nude mice bearing HepG2 cells	[92]
Res	Res-conjugated AuNPs	DD		breast cancer	TPA-induced migration and invasion in breast cancer MCF-7 cells		[93]
Res	Res-conjugated AuNPs stabilized by gum arabic	DD		cancer	breast cancer MDAMB-231, pancreatic cancer PANC-1, and prostate cancer PC-3 cells		[94]
Res Dox	Res-stabilized AuNPs	DD		brain cancer	human glioma LN 229 cells		[95]
Res Dox	AuNPs capped with Res	DD		cervical cancer	human cervical cancer (HeLa, HPV-18 positive, and CaSki, HPV-16 positive) cells		[96]
Res	Res-loaded in chitosan modified liposomes coated by gold nanoshells	NIR- and pH-responsive system	AuNPs Chitosan	cervical cancer	human epithelioid cervix carcinoma HeLa cells		[97]
Res	Hollow NPs based on Au-Res complexes	NIR responsive system	AuNPs	melanoma	malignant melanoma A375 cells		[98]

Res: Resveratrol; Au: gold; NPs: nanoparticles; AuNPs: gold nanoparticles; DD: drug delivery; Dox: doxorubicin; NIR: near infra-red; MRI: magnetic resonance imaging; TPA: 12-*O*-tetradecanoylphorbol-13-acetate.

**Table 4:** Hybrid systems based on AuNPs and Cur and projected as potential candidates for the treatment of human cancer.

Cytotoxic Drug	Hybrid Materials	Aim	Functionalization	Pathology	In Vitro Model	In Vivo Model	References
Cur	Cur-conjugated AuNPs	DD		cervical cancer	cervical cancer HeLa cells		[99]
Cur	Cur-capped AuNP-reduced graphene oxide nanocomposite	DD		colon and liver cancer	human colon cancer HT-29 and SW-948 cells		[100]
Cur	MUC-1 aptamer conjugated and Cur-loaded PEGylated amine-terminated generation 5 poly(amidoamine) dendrimers/gold hybrid structures	active TDD	MUC-1 aptamer	colon adenocarcinoma	colon cancer HT29 and C26 cells	C26 tumor-bearing BALB/c female mice	[101]
Cur Lipoic acid	Lipoic acid-Cur and GSH attached to gold-iron oxide nanocomposites	active targeted and pH-responsive DD MRI	GSH Lipoic acid Iron NPs	Brain cancer	fetal human astrocyte and U87MG cell lines		[102]
Cur	Cur-loaded gliadin-stabilized folic acid-functionalized Au quantum clusters	active targeted and pH-responsive DD	Folic acid Gliadin	Cancer	brain cancer C6 glioma cells and breast cancer MDA-MB231 cells		[103]
Cur	Cur-loaded in protein polymer-Au NPs (protein polymer based on elastin-like peptide and the coiled-coil region of Cartilage Oligomeric Matrix protein, both bearing an *N*-terminal hexahistidine group)			Breast cancer	human breast cancer MCF7 cells		[104]
Cur	Folate-Cur-loaded Au-polyvinylpyrrolidone NPs	active targeted DD	Folic acid	breast cancer	human breast adenocarcinoma MDA-MB-231 and MCF-7, epithelial MCF 10A cells; mouse mammary carcinoma 4T1 cells	female Balb/c mice bearing 4T1 cancer	[105]
Cur	AuNPs immobilized on folate-conjugated dendritic mesoporous silica-coated reduced graphene oxide nanosheets loaded with Cur	active targeted DD NIR- and pH-responsive system	AuNPs Folic acid	breast cancer	human breast adenocarcinoma MCF-7 cells		[106]
Cur	chitosan-coated halloysite nanotubes loaded with Cur-Au NPs	NIR- and pH-responsive systems	AuNPs Chitosan	breast cancer	human breast adenocarcinoma MCF-7 cells		[107]
Cur	Cur-loaded in gold-coated liposome NPs	NIR-responsive system	AuNPs	melanoma	mouse melanoma B16 cells	C57BL/6 female mice bearing B16 cells	[108]
Cur	Cur-loaded HSPC liposomes coated with gold	NIR-responsive system	AuNPs	melanoma	mouse melanoma B16 F10 cells		[109]
Cur	Cur-Au-PEG-NPs	NIR-responsive and sonosensitive system	AuNPs Ultrasounds	melanoma	mouse melanoma C540 (B16/F10) cells	inbred male BALB/c mice bearing B16/F10 cells	[110]
Cur	PEG-Cur-AuNPs	NIR-responsive system	AuNPs	melanoma	mouse melanoma C540 (B16/F10) cells		[111]
Cur	PEG-Cur-AuNPs	NIR-responsive system	AuNPs	melanoma	mouse melanoma C540 (B16/F10) cells	male C57/inbred mice implanted with B16/F10 cells	[112]
Cur	Cur-loaded Ag/Au bimetallic NPs coated with polystyrene- and PEG-based gel layers	NI- responsive system	AuNPs	melanoma	mouse melanoma B16F10 cells		[113]

Cur: curcumin; Au: gold; NPs: nanoparticles; AuNPs: gold nanoparticles; DD: drug delivery; TDD: targeted drug delivery; NIR: near infra-red; MRI: magnetic resonance imaging; HSPC: hydrogenated soya phosphatidyl choline; PEG: polyethylene glycol; GSH: reduced glutathione.

## Abbreviations

AuNP	gold nanoparticle
CTS	chitosan
Cur	curcumin
Cur@AuNP	Cur-containing AuNP
DDS	drug delivery system
DNMT	DNA methyltransferase
Dox	doxorubicin
DSS	dextran sodium sulfate
ECM	extracellular matrix
EGFR TK	epidermal growth factor receptor tyrosine kinase
ER	estrogen receptor
FA	folic acid
G	graphene
GO	graphene oxide
GSH	reduced glutathione
HSPC	hydrogenated soya phosphatidylcholine
LA	lipoic acid
Lip	liposome
MMP	matrix metalloproteinase
MRI	magnetic resonance imaging
MUC-1	mucin-1
NF-κB	nuclear factor-κB
NIR	near-infrared
NP	nanoparticle
PAMAM	poly(amidoamine)
PDT	photodynamic therapy
PEG	polyethylene glycol
PS	polystyrene
PTT	photothermal therapy
PVP	polyvinylpyrrolidone
Res	resveratrol
rGO	reduced graphene oxide
ROS	reactive oxygen species
SAR	structure-activity relationship
SDT	sonodynamic treatment
STAT3 protein	signal transducer and activator of transcription 3 protein
TDD	targeted drug delivery
TPA	12-*O*-tetradecanoylphorbol-13-acetate
TrxR	thioredoxin reductase
VEGF	vascular endothelial growth factor
